# Trends and Projections in Breast Cancer Mortality among four Asian countries (1990–2017): Evidence from five Stochastic Mortality Models

**DOI:** 10.1038/s41598-020-62393-1

**Published:** 2020-03-25

**Authors:** Sumaira Mubarik, Fang Wang, Muhammad Fawad, Yafeng Wang, Ishfaq Ahmad, Chuanhua Yu

**Affiliations:** 1 Department of Epidemiology and Biostatistics, School of Health Sciences, Wuhan University, Wuhan, Hubei 430071 China; 2 Henan Academy of Big Data, Zhengzhou University, Zhengzhou, 450052 China; 3 School of Mathematics and Statistics, Zhengzhou University, Zhengzhou, 450001 China; 4 Department of Mathematics and Statistics, Faculty of Basic and Applied Sciences, International Islamic University, Islamabad, Pakistan; 5 Global Health Institute, Wuhan University, Wuhan, Hubei 430071 China

**Keywords:** Health policy, Epidemiology, Epidemiology, Risk factors

## Abstract

The current study aimed to explore some important insights into the breast cancer mortality (BCM) trends and projections among four Asian countries by using five advanced stochastic mortality models. BCM data over 28 years from 1990–2017 with ages 20–84 were retrieved from the Global Burden of Disease (GBD) Study 2017 for four Asian countries, namely, China, India, Pakistan, and Thailand. Five stochastic mortality models with the family of generalized age-period-cohort were implemented to find the present and future BCM trends in these four Asian countries. Based on Cairns-Blake-Dowd (CBD) model and Lee-Carter model (LCM), overall, results revealed that BCM increased with the passage of time. Aging factor was the most influential factor of elevated BCM in each Asian country under consideration. Projection of BCM showed that mortality rates might continue to grow with time, especially in older ages in each Asian country under study. The highest forecasted BCM rates were observed in Pakistan as compared to other countries. The obvious increase in BCM suggested that earlier tactics should be implemented to reduce the subsequent morbidity and mortality due to breast cancer. The last but not least, some additional tactics to mitigate the BCM in older ages must be adopted.

## Introduction

Breast cancer (BC) is a mutual malignancy worldwide and the second foremost source of cancer- related morbidity and mortality among Asian women contributing approximately 40% of BC worldwide. Asia being the largest Continent on the earth carries approximately 60% of the human population. Asia shows higher mortality to BC incidence ratio than western countries^1,2^. According to a study conducted on BC differences in Asian regions, reported that BCM due to higher prevalence of BC risk factors like delayed childbirth, increased obesity and reduced parity increased among Asian women^3^. Currently, a study reported the high body mass index as a significant risk factor of increased BC mortality among Chinese women^4^. In developing Asian countries where BC is found at the older ages, mortality rate due to BC is higher than the Western countries^1,2^. Being the top ranked populous country of Asia, China accounts for 25% of the overall deaths due to cancer especially in the younger population^5,6^. Many studies on BC survey in China reported 36.1% of mortality increased from the 1970 to 2005^4,7^, inviting the researchers’ attention for in-depth study on this matter. India is the second populous country in the world with the evidence of BCM as 12.7 per 100,000 patients^2,8^. Pakistan is the seventh populated country worldwide and also included in the list of countries having high BC mortality rates. Various risk factors like economic, demographic, reproductive and social issues are contributing to an increased burden of BC. BC is a commonly diagnosed malignancy in Pakistan where one woman in every nine women is facing this brutal disease^6^. Thailand is also facing the increased cancer-related burden^9^. A study reported that women at age of 40 got diagnosed more BC incidence than younger ones due to various socioeconomic, reproductive and environmental factors in Thailand^10^.

Precise estimates of future mortality due to BC are of immense importance to make endorsements for the allocation of resources to BC control programs and health care centers. Various studies used Age–Period–Cohort (APC) models to estimate trends in mortality, BC incidence, cervical cancer, and prostate cancer^5,11,12^. Although, some studies employed functional data analysis and Lee-Carter models to forecast the brain, breast and colon cancer respectively^13–15^. Recently, trend estimation of mortality and BC incidence is a topic of interest^11,12,16,17^. However, limited literature is available on forecasting of its future trends^15,18^. Additionally, few signs of progress have been seen in developing and introducing advance statistical methodology for mortality forecasting due to cancer in general and BC in particular^19^.

In this study, we presented advanced approaches for mortality forecasting by utilizing BC data. These approaches include stochastic modeling of mortality related to the family of Generalized Age-Period-Cohort (GAPC). This study aims to explore insights into BCM trends and projections among four Asian countries using five Stochastic Mortality Models (SMM). To the best of our knowledge, previously no study is available on the projection of BCM using SMM in these Asian countries. The novelty of this study lies in achieving the following specific objectives:

- To examine the trends of BCM among four Asian countries using advanced SMM.
- To compare the performance of various models using different diagnostic measures.
- To forecast the mortality trends using the best candidate model for future policy implications in four Asian countries.

## Materials and Methods

### Data source

The BCM data of 28 years from 1990–2017 with ages 20–84 years were obtained from the Global Burden of Disease (GBD) Study 2017 for four Asian countries, namely, China, India, Pakistan, and Thailand. GBD studies are based on data retrieved from Institute for Health Metrics and Evaluation (IHME) (http://ghdx.healthdata.org/gbd-results-tool). IHME is the part of the University of Washington and an independent global health research foundation being responsible for maintaining and exchanging comprehensive registry data, surveys, censuses, and other health-related data to produce various disease estimates. GBD estimates include incidence, mortality, prevalence, year of life lost (YLL), year lived with disability (YLD), and disability adjusted life years (DALY) of each disease and injuries. These estimates are reported by time, location, age group, and gender. The purpose of the GBD study is to establish inclusive and analogous global health metrics. For China, original data were retrieved from literature, Vital Registration, China Vital Statistics-deaths, the Ministry of Health, Cancer Registry, WHO Mortality Database and some other sources. Whereas, for Thailand, the similar data were composed form IACR-Thailand national cancer registry, Chiang Mai cancer registry, and national cancer institute Thailand. On the other hand, in India and Pakistan due to lack of proper records, BC incidence and mortality data were collected from hospital-based registries, national cancer registry, WHO Mortality Database. Some of the records were also estimated from other countries with similar socio-cultural attributes.

### Analytical methods

In this section, we described the five SMM related to the family of generalized age-period-cohort (GAPC). These five models include Lee-Carter Model (LCM), Age-Period-Cohort (APC), Cairns-Blake-Dowd (CBD), Quadratic CBD model (M7), and cohort extension of the Lee-Carter model (RH). These models not only measure the effect of age, period, and birth cohort with different extension of these factors but also the past historical effects by forecasting mortality trends. Five models used in this study are based on the “extrapolative” forecasting method. This method is based on past mortality trends assuming that trends are repeating themselves in the future^20^. The models specification, their brief description and selection criteria are given in the next sections.

### Generalized age-period-cohort stochastic mortality models

Among the famous approaches of GAPC stochastic modeling, LCM model is of great interest for forecasting the mortality trends. Many researchers have developed several variants and extensions of LCM, for example; the use of generalized hyperbolic distributions or Poisson distribution settings for Lee Carter random components to tackle the assumption of normality^21^. Recently, LCM has been used to forecast the mortality rates for colon cancer and similar results obtained under Maximum Likelihood Estimation (MLE) and Singular Value Decomposition (SVD) settings^13^. To improve the goodness-of-fit as well as forecasting power the alternative estimation approaches are also proposed in various studies^22–24^. Some authors have extended the LCM by adding the two age period factor^25,26^. Original LCM is also being considered with the inclusion of cohort effect term^27^. A two-factor CBD model has also been developed for the mortality odds ratios. This kind of model depends on the linearity of the logit (odds). Recently, LCM and the CBD model are applied on Italian mortality data. The assessment of these two models lead to the conclusion that both CBD model and LCM projections are consistent generally for older ages (>75)^28^. Later, CBD model is extended by adding quadratic age term and a cohort effect term known as M7 model^29^.

### Lee carter model

The work of Lee and Carter (1992) is the significant contribution in mortality forecasting^30^. LCM is considered as scientific model, which does not require personal opinion or reasons for mortality. As it integrates mortality dynamics of age and time (in calendar years) and is based on the lag effect of age and period. The general statistical form of LCM is expressed as:

1 $$\mathrm{ln}({M}_{x,t})={\alpha }_{x}+{\beta }_{x}^{(1)}{k}_{t}^{(1)}+{\varepsilon }_{x,t}$$

where ${M}_{x,t}$ represents the mean BCM rate at age $x$ in year $t$, ${\alpha }_{x}$ shows the mean trend of BCM by age across the years, $\,{\beta }_{x}^{(1)}$ shows the slope in the mean rate of BCM concerning variations in ${k}_{t}^{(1)}$, obtained by differentiating the $\mathrm{ln}({M}_{x,t})\,$ at time $t$, ${k}_{t}^{(1)}$ represents BCM level index at time $t$, and ${\varepsilon }_{x,t}$ is the residual term which represents the random historical fluctuations not explained in the model. It is assumed that ${\varepsilon }_{x,t}$ is an independent and identically distributed (IID) Gaussian random variable with mean 0 and constant variance$\,({\sigma }^{2})$. Parameter ${\alpha }_{x}$ is based on an average $\mathrm{ln}({M}_{x,t})$ over time$\,t$. The unique solution of ${\beta }_{x}^{(1)}\,and\,{k}_{t}^{(1)}$ was obtained by imposing two constraints on parameters ${\beta }_{x}^{(1)}\,and\,{k}_{t}^{(1)}$ ^30^.

The ${\alpha }_{x}$ are mean of $\,\mathrm{ln}({M}_{x,t})$ over time $t$, ${\beta }_{x}^{(1)}$ and ${k}_{t}^{(1)}\,$sum to unity and 0, respectively. Using the result of these constraints the parameter ${\alpha }_{x}\,\,$ can be estimated by formula (2),

2 $${\hat{\alpha }}_{x}\,=\frac{{\sum }_{t=1}^{T}\,\mathrm{ln}({M}_{x,t})}{T}\,$$

Where, ${\hat{\alpha }}_{x}\,$ shows the mean of natural logarithm of BCM rates ($\mathrm{ln}({M}_{x,t})$) over time $t$ for individual aged $x$ for total number of period T. After estimating the parameter ${\hat{\alpha }}_{x}\,$for all values of age $x$ are subtracted from the LCM Eq. (1) to get ${\beta }_{x}^{(1)}$ and ${k}_{t}^{(1)}$. The MLE method provides biased estimates because of parameter constraints^31^. Therefore, remaining parameters were estimated using the SVD method^30,32,33^. The general procedure of SVD is as follow:

M1 $${Z}_{x,t}=[\begin{array}{llll}{\hat{\beta }}_{{x}_{1}}^{(1)}{\hat{k}}_{{t}_{1}}^{(1)} & {\hat{\beta }}_{{x}_{1}}^{(1)}{\hat{k}}_{{t}_{2}}^{(1)} & \ldots  & {\hat{\beta }}_{{x}_{1}}^{(1)}{\hat{k}}_{{t}_{T}}^{(1)}\\ {\hat{\beta }}_{{x}_{2}}^{(1)}{\hat{k}}_{{t}_{1}}^{(1)} & {\hat{\beta }}_{{x}_{2}}^{(1)}{\hat{k}}_{{t}_{2}}^{(1)} & \ldots  & {\hat{\beta }}_{{x}_{2}}^{(1)}{\hat{k}}_{{t}_{T}}^{(1)}\\ \vdots  & \vdots  & \ldots  & \vdots \\ {\hat{\beta }}_{{x}_{n}}^{(1)}{\hat{k}}_{{t}_{1}}^{(1)} & {\hat{\beta }}_{{x}_{n}}^{(1)}{\hat{k}}_{{t}_{2}}^{(1)} & \ldots  & {\hat{\beta }}_{{x}_{n}}^{(1)}{\hat{k}}_{{t}_{T}}^{(1)}\end{array}]\,$$

M2 $$SVD({Z}_{x,t})=ULV{\prime} $$

The ${Z}_{x,t}$is the matrix of order n × T that contains the estimates of parameters$\,{\beta }_{x}^{(1)}{k}_{t}^{(1)}$, further, the total number of ages and periods included in model are denoted by n and T. Applying SVD on ${Z}_{x,t}$ alternatively, M1 gives the product of three matrices U, L and $V{\prime}$, which is denoted as M2. Here, U represents the age factor matrix carrying the order n × n, L represents the diagonal matrix of singular values with order n × T, while $V{\prime}$matrix identifies the transpose of the time component matrix V with order T × T. The matrix M1 shows the estimates of ${\beta }_{x}^{(1)}$ and ${k}_{t}^{(1)}$ after the application of SVD.

### Age-period-cohort model

Age-Period-Cohort (APC) model is the extension of the LCM. The APC model was obtained by extending the LCM with addition of the cohort effect^27^. This additional cohort term (“period-age”) measures the observational effect i.e. those born in the similar cohort will likely to have an identical BCM trend. The mathematical expression of the APC model is as shown below:

3 $$\mathrm{ln}({M}_{x,t})={\alpha }_{x}+{\beta }_{x}^{(1)}{k}_{t}^{(1)}+{\beta }_{x}^{(2)}{\gamma }_{t-x}^{(2)}+{\varepsilon }_{x,t}\,$$

Where parameter ${\gamma }_{t-x}^{(2)}$ measures the effect of the cohort at aged $x$, and year $t-x$ is introduced to avoid the identification problem. Two additional restrictions $\sum _{x}{\beta }_{x}^{(2)}$ = 1 and ${\gamma }_{t-x}^{(2)}$ = 0 were imposed in the APC model while others were kept same as imposed in previous LCM.

### Cairns-Blake-Dowd model

Cairns *et al*.^34^ developed Cairns-Blake-Dowd (CBD) model by introducing age period predictor structure with two age-related modified parameters such as ${\alpha }_{x}=1$ and ${\beta }_{x}^{(1)}=(x-\bar{x})$ respectively. Overall, the model belongs to the family of Generalized Linear Models (GLM) with response variable as BCM odds, the ratio of BCM (${M}_{x,t}$) to survival (1−${M}_{x,t}$) rate. Model expression is as follows:

4 $${\rm{logit}}({M}_{x,t})=\,\log \left(\frac{{M}_{x,t}}{1-{M}_{x,t}}\right)={k}_{t}^{(1)}+(x-\bar{x}){k}_{t}^{(2)\,}$$

Where $\bar{x}$ represents the mean age, ${k}_{t}^{(1)}$ and ${k}_{t}^{(2)\,}$ are the two time-related parameters. Identifiability issue does not exist in the CBD model, therefore no need to impose the parameter constraints in CBD model. For parameter estimation of the CBD model, we can follow Haberman and Renshaw^35^ by assuming a Poisson distribution of mortality.

### M7 model

Quadratic CBD model with cohort effect is known as M7 model. It is an extension of the original CBD model with the addition of cohort effects and quadratic age effect. Cairns *et al*.^29^ developed this CBD extension and introduced the new predictor structure in CBD. The pre-specified quadratic age and cohort modulating parameters are $\,{\alpha }_{x}=1$, $\,{\beta }_{x}^{(1)}=(x-\bar{x})$, $\,{\beta }_{x}^{(2)}=({(x-\bar{x})}^{2}-\,{\hat{\sigma }}_{x}^{2})\,\,$and ${\beta }_{x}^{(3)}=1$. The model expression is as follows:

5 $${\rm{logit}}\,({M}_{x,t})=\,\log \left(\frac{{M}_{x,t}}{1-{M}_{x,t}}\right)={k}_{t}^{(1)}+(x-\bar{x}){k}_{t}^{(2)\,}+({(x-\bar{x})}^{2}-\,{\hat{\sigma }}_{x}^{2}){k}_{t}^{(3)\,}+{\gamma }_{t-x}$$

Where response variable BCM odds is represented by, $(\frac{{M}_{x,t}}{1-{M}_{x,t}})$, i.e. BCM (${M}_{x,t}$) to survival (1−${M}_{x,t}$) rate ratio, the mean value of ${(x-\bar{x})}^{2}$ is denoted by ${\hat{\sigma }}_{x}^{2}$, which shows the average variance. To solve the problem of identification, the following sets of constraints are imposed:

$$\sum _{x}{\beta }_{x}^{(2)}=1,\,\sum _{t}{k}_{t}^{(3)\,}=0,\,{\rm{and}}\,\sum _{x}{\beta }_{x}^{(3)}=1,\,{\gamma }_{t-x}=0\,{\rm{or}}\,\mathop{\sum }\limits_{c={t}_{1-{x}_{k}}}^{{t}_{n-{x}_{1}}}{\gamma }_{c}=0.$$

### RH model

RH model is the generalized form of the LCM, with the addition of the cohort effect. It developed by Renshaw and Haberman^27^ and recognized as RH model. The statistical form of the model is as follow:

6 $$\mathrm{ln}\,({M}_{x,t})={\alpha }_{x}+{\beta }_{x}^{(1)}{k}_{t}^{(1)}+{\beta }_{x}^{(0)}{\gamma }_{t-x}$$

Where, ${M}_{x,t}\,$represents the mean BCM rate at age $x$ in year $\,t$, ${\alpha }_{x}$ shows the mean BCM trend by age, $\,{\beta }_{x}^{(1)}$ indicates the mean slope in the central BCM rate concerning fluctuations in ${k}_{t}^{(1)}\,$and ${\beta }_{x}^{(0)}\,$represents the mean trend of BCM by cohort across the age $\,x$. For a unique solution of the parameters, the following restriction was imposed:

$$\sum _{x}{\beta }_{x}^{(1)}=1,\sum _{t}{k}_{t}^{(1)}=0,\sum _{x}{\beta }_{x}^{(0)}=1,\mathop{\sum }\limits_{c={t}_{1-{x}_{k}}}^{{t}_{n-{x}_{1}}}{\gamma }_{c}=0.$$

BCM forecast of RH model is obtained by forecasting of estimated components ${k}_{t}^{(1)}\,$and ${\gamma }_{t-x}$. These components are generated by autoregressive integrated moving average (ARIMA) process with the assumption of no dependence between period and cohort. The distribution of BCM rates (${M}_{x,t}$) is assumed as Poisson to estimate the parameters.

### Performance measures

The performance measures include Mean Error (ME), Root Mean Squared Error (RMSE), Average Root Mean Squared Error (ARMSE), Mean Absolute Error (MAE), Mean Absolute Percentage Error (MAPE) and Residual analysis. These are used to determine the best fit of the model. The Mean Error (ME) is the difference between actual and fitted values. It can produce biased results. While RMSE is considered more reliable measure as it eliminates the drawback of the ME by taking into account the square of differences between actual and fitted values^36^. Therefore, in this study, we used RMSE and ARMSE. Minimum RMSE indicates the excellent performance of the model with the explanation that all the required information available in data. For proper model fitting, residuals should be independent and identically distributed with zero mean. This assumption may be tested by graphical analysis. The residuals scatter plots versus age, calendar year, and year of birth are used to check the validity of this assumption. Lack of randomness in the residuals patterns indicates the inability of a model to capture specific age, period, or cohort effects. Moreover, the prediction interval (PIs) of future BCM rates is also used to check the consistency of the estimates. The narrow width of the prediction interval leads to the precision of such estimates.

## Results

### Descriptive analysis of BCM

**Figure 1:**
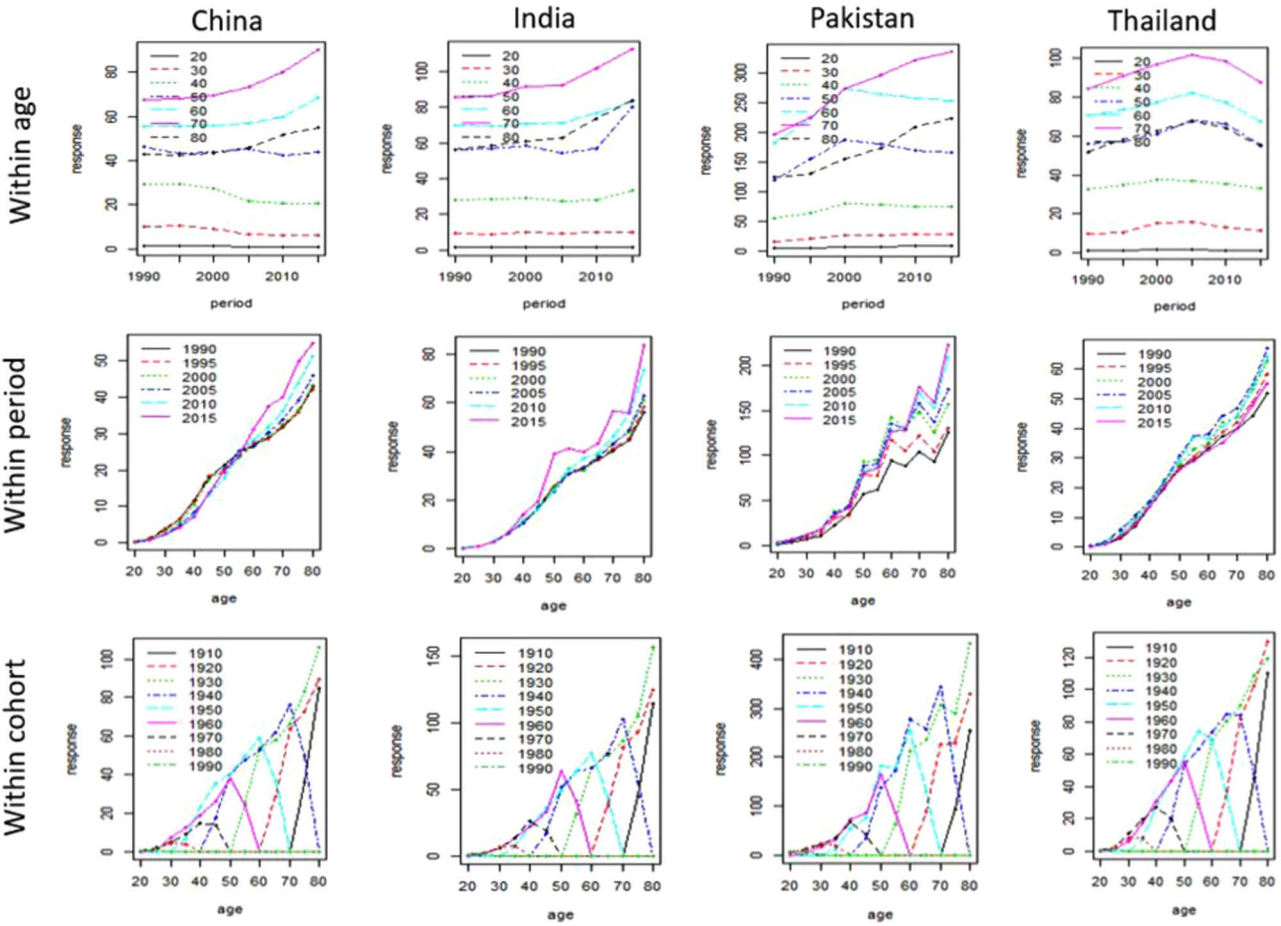
Trends of BCM rates by age, period, and cohort for four Asian countries from 1990 to 2017. response: BCM rates (per 100,000).

Figure 1 shows the trends in BCM among four Asian countries women for age, period, and cohort between 1990 and 2017. In all Asian regions including China, India, Pakistan and Thailand, lower BCM rates are observed for young women (age ≤ 40 years). The rates were higher in 2015 for older women (age > 50 years) in all Asian countries under study. Moreover, higher BCM was also noticed within the early cohorts (1950 and below).

**Figure 2:**
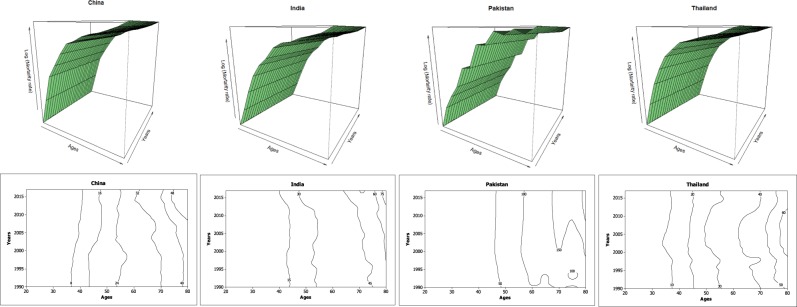
Growth of BCM in Asian women separately for each country aged 20 to 84 for the years 1990 to 2017.

On the other hand, BCM rates were seen to be sparse for young women especially in recent cohorts. We used the response surface and contour plot to depict the increase in BCM of four Asian countries. The said plot was used to represent the BCM across ages and years simultaneously. From response surfaces and contours, it can be seen that the BCM value was lower before 2005, and gradually increased until the end of the year. Subsequently, after fifty years of age, the response surface supported the gradual mounds along the years and BCM increased steadily for older women. Although, these continuous mounds tend to appear as little random sequence in Pakistani women but still maintain a higher rate of BCM as compared to other Asian countries (Fig. 2).

### Estimation of breast cancer mortality trends using various models

**Figure 3:**
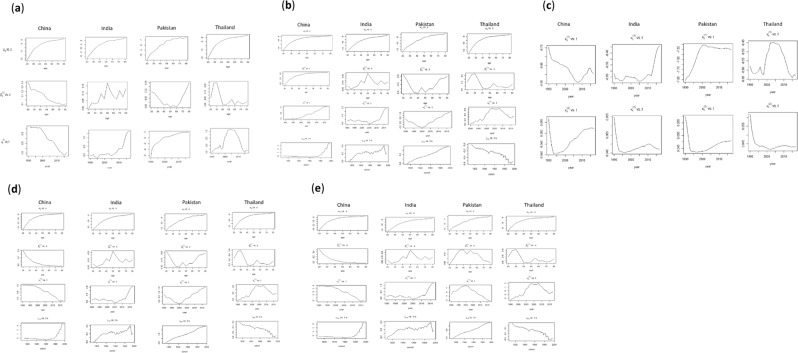
Estimation of the (**a**) LCM (**b**) APC (**c**) CBD (**d**) M7 and (**e**) RH model parameters for four Asian countries.

BCM rates from 1990 to 2017 for women with ages 20 to 84 years were considered to fit LCM by applying SVD estimation method. In Fig. 3(a), the parameter $\,{\alpha }_{x}$ displays the general BCM trend across ages $x$ for four Asian countries. The upward trend of BCM is observed in $\,{\alpha }_{x}$ for all the mentioned countries showing that women with younger ages carry lower BCM than older ones. Whereas, ${\beta }_{x}^{(1)}$ represents the BCM change over the altered age $\,x$. It is depicting an almost linear downward trend for Chinese women with several jumps. An increased systematic pattern is being perceived among 50-year-old Indian women carrying the highest rate. A roughly U shaped trend was comprehended among Pakistani women. It represents the higher rate of change in older ages. In Thailand, an increasing trend of ${\beta }_{x}^{(1)}$ is also observed at younger age (20–35), and then downward trend where it falls sharply. After the age of 40 years, ${\beta }_{x}^{(1)}$ has been seen as invariant for all the following ages. The ${k}_{t}^{(1)}\,$shows the changes in the BCM index over time. Almost constant and highest rate of change was observed during 1990–2000, which gradually decreased until 2015, and then it started to increase among Chinese women. An increasing trend was observed among Indian women from 2005 to 2017. Whereas, higher and steadily growing pattern of change was found among Pakistani women throughout the period. From 1998 to 2015, the variation in BCM for Thailand women was seen alike quadratic shape.The estimated trends of$\,{\alpha }_{x}$, ${\beta }_{x}^{(1)}$ and ${k}_{t}^{(1)}$ by APC model were similar to the trends of LCM. It has the same interpretation as LCM and consequently reveals the same shape to these parameters for all mentioned countries. Additionally, patterns of cohort effect ${\gamma }_{t-x}$ across the cohort, $t-x$ shows a linear trend of change from early to later cohorts. For China, it can be seen that women born in 1960–1964 were observed to be at lower risk than those born in 1955–1959 or before. While, for India, the women born in cohort 1920–1950 carried higher risk as compared to subsequent cohorts, with the exception of cohort 1990 with higher rate. For Pakistani women, the gradually increasing effect was observed throughout the cohort. The progressively decreasing trend of change was observed in early to later cohorts among women of Thailand (Fig. 3(b)).

Figure 3(c) shows the changes in BCM by CBD model with time-dependent parameters of four Asian countries. For China, the first estimated parameter ${k}_{t}^{(1)}$ showed a downward trend over time from 1990 to 2007, and later increased sharply until 2015. For India, the invariant trend of the BCM index was observed over time despite of experiencing a few jumps until 2013. However, for Pakistan, BCM index showed a sharp increasing trend from 1990 to 2000. Afterward, it began to decline slightly until the end of the period. Whereas, for Thailand, the trend of ${k}_{t}^{(1)}$ showed almost similar patterns as noticed in the LCM and APC. The second time-dependent BCM change parameter $\,({k}_{t}^{(2)})\,\,$ of the CBD model measures the rate of change in BCM improvements. The change in BCM for all Asian countries was observed at peak level in year 1990 (Fig. 3(c)).

The estimated trends of $\,{\alpha }_{x}$, ${\beta }_{x}^{(1)}$, ${k}_{t}^{(1)}$ and ${\gamma }_{t-x}$ using the M7 model were almost similar to APC. Therefore, these estimated trends have the same interpretation and thus exhibit a similar pattern for all mentioned countries. The trends of ${\beta }_{x}^{(1)}$ and ${k}_{t}^{(1)}\,$for China have some exception where they show an opposite trend to the APC model (Fig. 3(d)).

Figure 3(e) illustrated the evolution of the four age and time-dependent parameters such as $\,{\alpha }_{x}$, ${\beta }_{x}^{(1)}$, ${k}_{t}^{(1)}$ and ${\gamma }_{t-x}$ of the RH model for the Asian countries under consideration. The estimated trends of $\,{\alpha }_{x}$, ${\beta }_{x}^{(1)}$, ${k}_{t}^{(1)}$ and ${\gamma }_{t-x}$ by RH model was also similar as of APC and M7 models. The estimated parameters for these countries can be interpreted in the same way as for APC and M7 models, with exception of trend of ${\beta }_{x}^{(1)}$ and ${k}_{t}^{(1)}\,$for Pakistan. The trends of these two parameters show an opposite direction as compared to previous models. (Fig. 3(e)).

Supplementary Figure S1 (a) and (b) illustrated the plots of residuals versus age, period, and the cohort being extracted from the LCM model and CBD model respectively for each Asian country under consideration. It could be seen from Fig. S1(a) that maximum residuals exist near to zero clusters. This closeness demonstrates that residuals follow a normal distribution. On the other hand, some residuals were away from zero clusters. For example, residuals parallel to the ages 30–45 for China, 50–80 for India, 50–60 for Pakistan, and 30–60 years for Thailand. This dispersion identified that there were some outliers in the data. In Fig. S1(b), the maximum residuals were close to zero cluster. This feature depicted that the excellent fit of the CBD model for four Asian countries. Moreover, it was also observed that most of the residuals corresponded to the ages close to each other, which showed a minimum sum of the square of the residuals. Overall, the both models performed well for each Asian country. Here we have presented only limited comparison and included residual plot for two best-fitted models. A detailed comparison of all the study models have been discussed in the next section.

### Comparison of models performance

Supplementary Figure S2 (a) and (b) compared the root mean square error (RMSE) of five methods for each age and year, respectively. Overall, LCM, CBD, APC, M7, and RH models had quite comparable results after the age of 35. We noticed that, with lowest RMSE, the CBD model had smaller and more stable errors measures over the ages. It indicated that RMSE of CBD model was relatively more consistent across the ages as compared to other methods. Moreover, Fig. S2 (b) depicted the comparison of RMSE of five models over the years. The APC and RH for China, APC, LCM, M7, and RH for India, and APC, M7, and RH for Pakistan and Thailand showed a similar trend in terms of RMSE. Errors of the CBD model not only remained lower but also consistent across the year for each Asian country. Therefore, from these findings, the CBD model was considered as the most suitable for each population under consideration.

Tables 1 and 2 contained the average root mean square error (ARMSE) values for ages and time, respectively. The ARMSE over ages indicated that there was an insignificant difference between the methods (p > 0.05). ARMSE over time showed a significant difference among the methods (p < 0.001). We also observed that, concerning the lowest ARMSE, the CBD model ranked first best fit model for each Asian country. While, LCM model ranked second-best fit for each Asian country, except for Thailand. For Thailand, the LCM model showed a quite massive ARMSE as compared to the other models due to some outliers in data.

**Table 1:** Average root mean square error (ARMSE) of five methods over the ages 20–84 separately for four Asian countries.

Countries	Stochastic Mortality-GAPC Models				
	LCM (95% EI)	APC (95% EI)	CBD (95% EI)	M7 (95% EI)	RH (95% EI)
China	9.24 (8.21–10.26)	9.37 (8.09–10.64)	**8.87 (8.32–9.43)**	9.45 (8.31–10.58)	9.37 (8.09–10.64)
India	8.94 (7.98–9.90)	9.45 (8.31–10.58)	**8.63 (8.06–9.20)**	9.45 (8.31–10.58)	9.45 (8.31–10.58)
Pakistan	7.82 (6.96–8.68)	8.83 (7.63–10.02)	**7.55 (6.99–8.11)**	8.83 (7.63–10.02)	8.83 (7.63–10.02)
Thailand	9.17 (8.00–10.33)	9.17 (8.00–10.33)	**8.56 (8.03–9.08)**	9.17 (8.00–10.33)	9.17 (8.00–10.33)

Note: EI = 95% Error Interval.

**Table 2:** Average root mean square error (ARMSE) of five methods over the years 1990–2017 for four Asian countries.

Countries	Stochastic Mortality-GAPC Models				
	LCM (95% EI)	APC (95% EI)	CBD (95% EI)	M7 (95% EI)	RH (95% EI)
China	9.38 ^A^ (9.34–9.42)	9.58 ^A^ (9.41–9.74)	8.92^B^ (8.90–8.94)	9.13^B^ (9.10–9.16)	9.57 ^A^ (9.40–9.74)
India	9.07^B^ (9.05–9.09)	9.13 ^A^ (9.10–9.16)	8.67 ^C^ (8.64–8.70)	9.13 ^A^ (9.10–9.16)	9.13 ^A^ (9.10–9.16)
Pakistan	7.93^B^ (7.87–7.99)	8.44 ^A^ (8.39 8.48)	7.60 ^C^ (7.55–7.64)	8.44 ^A^ (8.39–8.48)	8.44 ^A^ (8.39–8.48)
Thailand	9.57 ^A^ (9.40–9.73)	8.91^B^ (8.87–8.95)	8.59 ^C^ (8.56–8.62)	8.91^B^ (8.87–8.95)	8.91^B^ (8.87–8.95)

Note: Means that do not share a letter are significantly different, EI = 95% Error Interval.

Furthermore, Post hoc analyses including Tukey HSD multiple comparison tests were performed to test the significant mean difference of ARMSE between various methods. Table 3 included the p-value of all possible pairs for each country. Results showed that, overall, the CBD model had the least error difference, and may be considered as appropriate fit for BCM data in this study. Comparing with other methods, only CBD and LCM model outperformed over all other models for India, Pakistan, and Thailand data (p < 0.001).

**Table 3:** Tukey HSD, Multiple comparisons of Average RMSE, mean difference p-values.

	China				India				Pakistan				Thailand			
	LCM	APC	CBD	M7	LCM	APC	CBD	M7	LCM	APC	CBD	M7	LCM	APC	CBD	M7
APC	0.082				0.048*				0.000**				0.000**			
CBD	0.000**	0.000**			0.000**	0.000**			0.000**	0.000**			0.000**	0.000**		
M7	0.016*	0.000**	0.055		0.048*	1.000	0.000**		0.000**	1.000	0.000**		0.000**	1.000	0.000**	
RH	0.089	1.000	0.000**	0.000**	0.048*	1.000	0.000**	1.000	0.000**	1.000	0.000**	1.000	0.000**	1.000	0.000**	1.000

Note: *p values are significant at 5% level of significance for that pair, **p values are significant at 1% level of significance for that pair.

### Forecasts of BCM rates based on selected models

**Figure 4:**
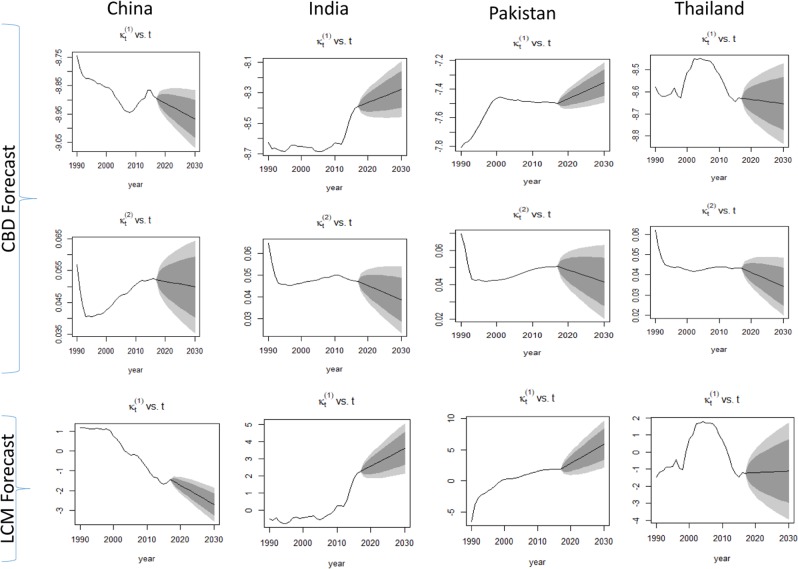
Forecast of the BCM index using the CBD model and LCM model for (1990–2030) period by countries. Shades represent 80% and 95% prediction intervals.

Figure 4 depicted the forecasts of the BCM indices by CBD and LCM models during the period 1990–2030 for four Asian countries. It was observed that the forecast pattern of ${k}_{t}^{(1)}$ vs time was similar by CBD model as well as by LCM for each Asian country. The overall trend predicted by the two models showed an increase in BCM trends for the period from 2018 to 2030 in India and Pakistan. Whereas, the forecast trend of BCM in China and Thailand showed a slightly declined trend for BCM. It indicated that the BCM was likely to decline in the future for both of these countries. However, it was also observed that the confidence interval of BCM for Thailand was wider than China. This was an indication of more uncertainty during that period.

**Figure 5:**
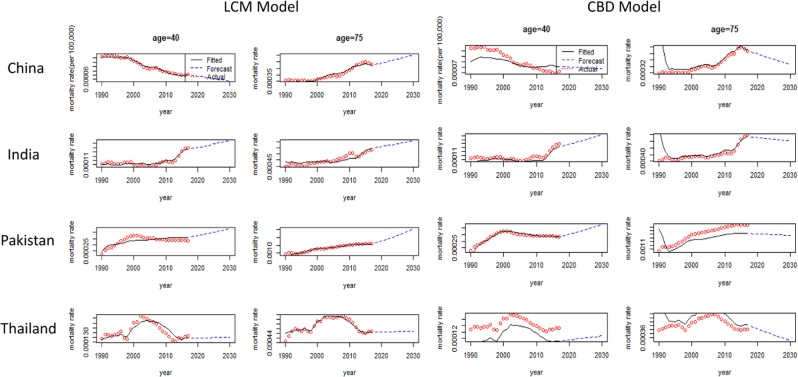
Forecast of the BCM rates (per 100 000) using the LCM and CBD model for (1990–2030) period by age and countries. Actual rate (red dot **◦**), fitted rate (black line-) and forecast rate (blue dashed line–).

Forecast pattern of ${k}_{t}^{(2)}$ vs time by CBD model represented the BCM change over time. It showed a slightly declined trend with time keeping a wide confidence interval for China and Pakistan. Figure 5 showed the fitted and projected BCM rates for the lower and upper age groups of each Asian country. We observed that for lower ages, fitted and projected BCM index showed a decreasing trend for China. On the other hand, for older ages, the predicted BCM index showed an upward trend in the LCM. Overall, each Asian country showed the chance to increase BCM in the future, particularly in older ages with different rates of BCM.

## Discussion

The present study provided an independent analysis of long-term BC mortality trends of women with ages from 20 to 84 years in Pakistan, China, Thailand, and India from 1990 to 2017. In our study, we considered different stochastic mortality models with the family of GAPC using SVD estimation algorithm. We also had used the residual analysis and Post Hoc analysis of RMSE and ARMSE to test and compare the performance of five models by age and time for each Asian country. Further, we also studied the trends of BCM across age, time, and birth cohort.

### Age, period and cohort related BCM trends

Generally, lower BCM rates were observed for young women (age ≤ 40 years) in all countries under consideration. On the other hand, higher rates were also seen in 2015 for old ages (>50 years) in Chinese, Indian, and Pakistani women. These results suggested that women with older ages contributed significantly to the increased BCM rates. Moreover, higher BCM was being noticed within the early cohorts (1950 and below), suggesting the positive association of BCM and cohort with birth before 1950.

This increased BCM trends are likely to be the reason due to insufficient access to proper BC treatment and disease control programs. Our findings are consistent with previous studies in different Asian countries^3,11,12^. Proper access to treatment facilitates and advanced treatments are likely the reasons for the decline in mortality in China. This may include improved early detection along with effective treatment. Most women under 50 years of age working in urban areas have employer-sponsored benefits like free medical examinations such as breast ultrasounds once or twice a year. Previous studies have shown that ultrasound is superior to Chinese women’s mammography for the prevention and control of BC^37^. Similarly, due to the improvement of modifiable risk factors, BC mortality rates have been decreased among younger women in developed countries such as the United States due to improvements in modifiable risk factors^38^.

However, the downward trends in India and Pakistan might be due to higher pregnancy rates and related reproductive factors^6,39^. In addition, lack of use of oral contraceptives is also likely to be the possible reason for the lower BCM rates in younger women^11^. Increased BCM in Thailand might be due to adopting a westernized lifestyle, various socio-demographic and environmental risk factors.

### Estimation and evaluation of stochastic mortality models for breast cancer data

The stochastic mortality models (SMM) belong to a standard age period cohort (APC) framework. It is a new approach to model age-specific mortality levels. All SMM have significant applications in different scientific fields. The main advantage of the stochastic mortality models is that in estimating and forecasting the mortality trends we do not need assumption of constant variation in mortality. Secondly, It also provides individual age-specific and period estimates^40^. Besides, these models can also provide individual residuals plot against age, time and cohort groups. These plots are very helpful to assess best fit of the model. Various studies suggest different evaluation criteria for comparison of SMM. In this study, we have considered RMSE, ARMSE and random residual pattern for best fit selection. The SMM has been used rarely in the field of epidemiology for modeling of BCM data. In this study, we introduce these methods and combine the knowledge of interdisciplinary fields to reflect the epidemiological characteristics of the disease. At the same time, we also attempted to promote the application of the methods by integrating social factors with an application in the field of public health. This study provides a new direction to estimate the BCM data more rationally and accurately. The Asian countries selected in this study are suffering from increasing BC burden and also represent similar circumstances related to over population, poverty, socio-cultural background, and insufficient access to diagnosis, advanced screening and proper treatment.

Limited studies are available on application of SMM to study mortality rate due to some specific cause. For example, Plevritis *et al*.^41^, used the SMM to estimate the age-specific BCM trends among U.S women. They reported declined BCM trends due to screening mammography and adjuvant therapy. Similarly, some Asian countries, investigated cancer incidence and mortality trends using APC and Joint point regression Model^11,42,43^. However, systematic analysis of BC mortality using stochastic mortality models like LCM, APC, CBD, M7, and RH model among these Asian countries under consideration has not been reported earlier.

In this study, five SMM models were estimated by using SVD algorithm and evaluated using the above-described criteria. A comparison of the five models over ages showed an insignificant difference among ARMSE for all models. While comparison over the years revealed a significant difference among the performance of five models using RMSE and ARMSE (p < 0.001). These results indicated that overall, the CBD model performs well for each Asian country. Many studies have been conducted where CBD model reported as the best fit for ages 55 and above for different diseases^29,34,44,45^. LCM model was considered to be the second-best fit model for each Asian country excepting for Thailand. On the other hand, the random pattern of residual plots against age, period and cohort, BCM data followed best fit using CBD and LCM models. This random pattern of residual plots advocated that these models can capture the age, period and cohort effects effectively.

Application of GAPC stochastic mortality models on BCM data provides the estimates of ${\alpha }_{x}$, ${\beta }_{x}^{(1)}$ and ${k}_{t}^{(1)}$ for LCM. Overall, ${\alpha }_{x}$ presented an upward trend against ages for each mentioned Asian country indicated that younger ages had lower BCM than older ones. It suggested that older age was strongly associated to increase BCM. Many other studies also reported the age as a substantial risk factor of increased BCM^3,12,46,47^. It shows the validity of ${\alpha }_{x}$ estimates by the LCM. Elhassan and Hamza pointed out the similar findings in a study on colon cancer^13^. On the other hand, ${\beta }_{x}^{(1)}$ exhibited an almost downward linear trend with few jumps for Chinese women. Increased systematic pattern of these values was observed among Indian women with the highest rate at age of 50 years. Almost U shaped trend was observed among Pakistani women, which represented the highest rate of change in older ages. Bray *et al*.^2^ also identified the increased mortality in older ages for both countries. In Thailand, almost increasing trend of ${\beta }_{x}^{(1)}$ was observed in younger ages (20–35) and thereafter sharp downward trend was seen. Further, after 40 years of age the values of ${\beta }_{x}^{(1)}$ start to increase despite of experiencing few jumps. Kim *et al*. also reported upward trends in BCM at age of 40 years and above in Thailand^3^. The ${k}_{t}^{(1)}\,$presented an increasing trend in recent years among Chinese women. While, gradually increasing trend was also shown among Indian women from 2005 to 2017. Whereas, higher and steadily increasing trend of mortality change was perceived among Pakistani women throughout the period. Larger rate of change in mortality indices was observed in 2015 for Thailand. The GLOBOCAN report 2018 also described the similar trend in recent years in Asian countries^2^. Moreover, in line with existing literature some Asian countries including China, India, Pakistan and Thailand, did not have much success in disease diagnosis and treatment programs as compared to some developed Asian and European countries during the developmental period^11,17,48^.

Results from the CBD model were illustrated in terms of two time-dependent parameters ${k}_{t}^{(1)}$ and ${k}_{t}^{(2)}$. These parameters represent trend and rate of change and are used to measure the fluctuations in mortality over time. For China, the first estimated parameter ${k}_{t}^{(1)}$ was seen to be increasing from 2007 to 2015. For India, the invariant trend of mortality index was observed over time with few jumps until 2013 and later it was increased sharply. While, for Pakistan, a sharp increasing trend of mortality index was observed over 1990 to 2000. Whereas, for Thailand, the trend of ${k}_{t}^{(1)}$ shows an almost similar pattern as noticed in the LCM framework. In each Asian country, a gradually increasing trend was seen after 1990, regardless of the small decrease in mortality until the end of the period. The decreasing trend of BCM may be due to the development of public health policies and improvements in treatment options. China is the most developed of the selected Asian countries, but its health status remains patchy and this phenomenon is more dominant in India and Pakistan. Over time, new advancements have been made in the health sector, but much remains to be done. Some prior studies conducted in China and Thailand had also reported similar kind of results^11,42,49,50^.

### Future prediction of BCM

Forecast pattern of ${k}_{t}^{(1)}$ versus time was quite similar under CBD and LCM for each Asian country. Increasing trends in BCM and BC incidence were already recounted in China and Thailand using APC and joint-point regression model in some previous studies^42,49^. However, in the current study, it is observed that the confidence interval to forecast BCM for Thailand was wider than China, which indicated more uncertainty during that period. Forecast pattern of ${k}_{t}^{(2)}$ versus time by CBD model reported the mortality change over time. It indicated slightly downwards trend over time but with wide confidence intervals for China and Pakistan. Therefore, the lower and upper age-wise pattern of mortality projection was depicted in Figure 5. It exposed the fitted and projected mortality rate in lower and upper ages for each Asian country. Projected mortality index for lower ages showed a declining trend in China. An increasing tendency of projection was observed for the upper ages by the LCM model for each Asian country. A study conducted by Cheng *et al*. also reported similar BCM trends in China^42^. Increased BCM in Thailand may be due to owing a westernized lifestyle and various socio-demographic and environmental risk factors. Overall, each Asian country under study has the chance to increase mortality in future, particularly in older ages with different rates of change in each region. Similar projections were reported for some other Asian countries^1,47,51,52^. Therefore, it may be concluded that CBD and LCM model can well explain the information from BCM data. Moreover, this study also reported that period effect played an essential role in BCM improvement. Therefore, it is urgently needed to adopt the appropriate strategies to reduce the current and future burden of morbidity and mortality due to specific cause breast cancer.

## Limitations

Regarding the study constraints, firstly, the data used in the study was based on the latest data from GBD 2017. Mortality data from the vital registration system and verbal dissections can also produce estimates of disease burden. However, diagnostic accuracy for the cause of mortality data is still limited, which may cause data accuracy issues. Although the results may vary to some extent from the original scenario. It is better to give at least a perspective of the diagnosis of the disease and its worst effects in the form of mortality. This will arouses the government and local bodies to maintain accurate records and ultimately requirements for disease diagnosis and management.

Secondly, further studies are needed to explain the some more characteristics of the selected models and justify scope of these models as compared to other models for BCM data. In spite of some limitations, our study is an essential and novel study countrywide. This study presents the application of advanced stochastic mortality models and investigation of mortality trends and future projections from specific cause breast cancer for four Asian countries.

## Summary

In short, five stochastic mortality models with the family of GAPC were estimated using SVD algorithm for breast cancer mortality (BCM) data. Estimated models were evaluated by residual analysis and Post Hoc analysis using RMSE and ARMSE for each Asian country data set. Based on the evaluation criteria CBD and LCM model follow the best fit of the BCM data. Results showed that breast cancer mortality is increasing with time. Older ages were observed as the most crucial factor influencing BCM. The period effect was more significant to rise the BCM trend in recent years. Projection of BCM showed that BCM might continue to increase with time, especially in older ages. The highest current and future forecast mortality trends were found in Pakistan as compared to other Asian countries. Therefore, the evident increase in BCM recommended that earlier tactics must be implemented to reduce the subsequent morbidity and mortality from specific cause breast cancer. The last but not least, additional tactics for dropping BCM in older ages also need to be strengthened in four Asian countries.

## Supplementary information

Supplementary information 

## Data Availability

The dataset analyzed during the current study are available in the Institute for Health Metrics and Evaluation (IHME): http://ghdx.healthdata.org/gbd-results-tool.
