# Supplementary material for: Trends and Projections in Breast Cancer Mortality among four Asian countries (1990–2017): Evidence from five Stochastic Mortality Models
Source: Sci Rep. 2020 Mar 25;10:5480. doi: 10.1038/s41598-020-62393-1 (PMC7096499; doi:10.1038/s41598-020-62393-1)
Supplement: Supplementary file 1 — Supplementary information [file 41598_2020_62393_MOESM1_ESM.pdf]

Trends and Projections in Breast Cancer Mortality among four Asian countries (1990-2017): Evidence from five Stochastic Mortality Models

Sumaira Mubarik, Fang Wang, Muhammad Fawad, Yafeng Wang, Ishfaq Ahmad and Chuanhua Yu*

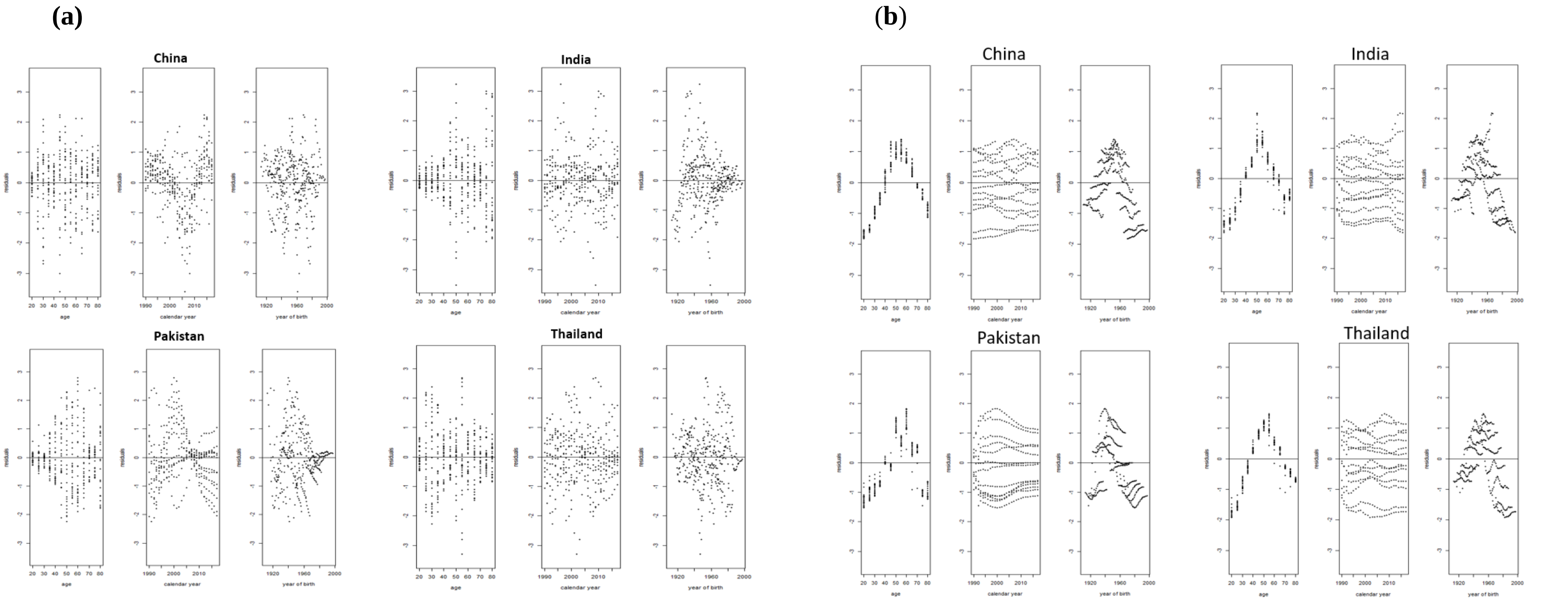

Figure S1. Plot of (**a**) LCM residuals against age, calendar year, year of birth, and (**b**) CBD residuals against age, calendar year and year of birth, for four Asian countries

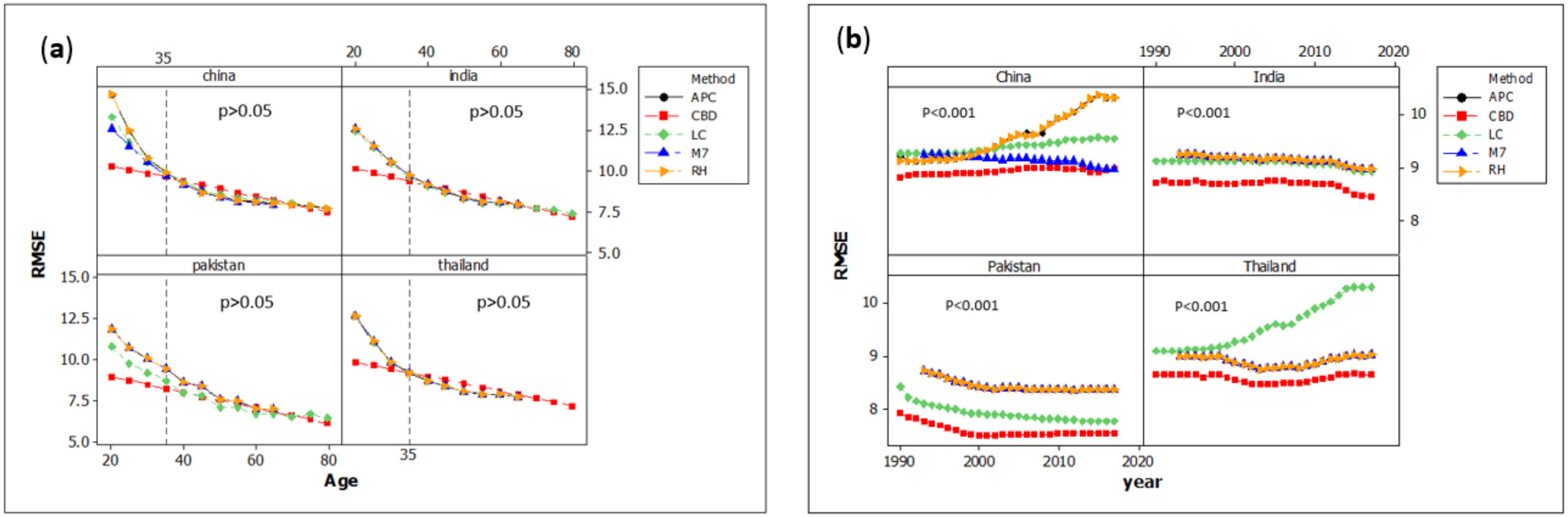

Figure S2. Plot of RMSE against (**a**) age for the overall population of 1990-2017 and (**b**) year for the overall population of 20-84 year of age, separately for each Asian country. Model were compared, and p-value obtained using F-test (ANOVA)
